# Perceived barriers to and suggested interventions for physical activity during pregnancy among participants of the Special Supplemental Nutrition Program for Women, Infants, and Children (WIC) in Southern California

**DOI:** 10.1186/s12884-021-03553-7

**Published:** 2021-01-21

**Authors:** Maria Koleilat, Nancy Vargas, Victoria vanTwist, Gergana Damianova Kodjebacheva

**Affiliations:** 1 Department of Public Health, College of Health and Human Development, California State University, 800 N. State College Blvd, Fullerton, CA 92831 USA; 2 College of Public Health and Human Sciences, Oregon State University, 160 SW 26th St, Corvallis, OR 97331 USA; 3 Department of Public Health and Health Sciences, College of Health Sciences, University of Michigan – Flint, 303 E. Kearsley St., Flint, MI 48502 USA; 4 International Institute, University of Michigan – Ann Arbor, Ann Arbor, MI USA

**Keywords:** WIC, Physical activity, Pregnancy, Focus group

## Abstract

**Background:**

The American College of Obstetricians and Gynecologists (ACOG) recommends that pregnant women engage in at least 20 to 30 min of moderate-intensity physical activity on most days of the week. Regular exercise during pregnancy is associated with many benefits for the mother and the developing fetus; yet, a large number of pregnant women do not engage in the recommended amounts. This study aimed to investigate barriers to and interventions for physical activity among pregnant WIC participants in Southern California.

**Methods:**

We conducted four focus groups (FGs) with pregnant low-income women aged 18 years or older in either their second or third trimester. FGs were conducted at a WIC center in Southern California. The FGs were held according to language (English vs. Spanish-speaking) and BMI category (normal weight vs. overweight and obese). A total of 28 women participated. We used ATLAS. ti. to analyze the focus group transcripts. The study adhered to the Consolidated Criteria for Reporting Qualitative Research.

**Results:**

The mean age of focus group participants was 28.9 years (SD = 6.6), and the majority were Latina. Intrapersonal barriers to physical activity were fatigue and lack of energy, pain and swelling, lack of childcare, medical restrictions and safety concerns, lack of knowledge about exercise safety, and lack of time. Interpersonal barriers included concerns and lack of support from partners and families, conflicting advice from friends and neighbors, and lack of advice on safe exercise from physicians. Women in all four groups suggested a community-based intervention where they can mingle with each other and share their challenges and concerns. Other suggestions to interventions differed among groups and reflected the women’s experiences and backgrounds. Specifically, to promote education, English-speaking women preferred a brochure while Spanish-speaking women preferred a video. Overweight women emphasized including children in their exercise activities to promote healthy behavior in youth.

**Conclusions:**

Interventions should be tailored to pregnant women’s needs. Primary care providers should provide reassurance and information to pregnant women and their partners on the type and frequency of safe exercise.

**Supplementary Information:**

The online version contains supplementary material available at 10.1186/s12884-021-03553-7.

## Background

The American College of Obstetricians and Gynecologists (ACOG) recommends that pregnant women engage in at least 20 to 30 min of moderate-intensity physical activity on most days of the week [1, 2]. Regular exercise during pregnancy is associated with many benefits for the mother and the developing fetus; yet, a large number of pregnant women do not engage in the recommended amounts [3, 4]. In fact, physical activity declines as the pregnancy progresses [5]. A study by Chasan-Taber et al. [6] showed that women who did not meet physical activity recommendations in late pregnancy had higher gestational weight gain (GWG) than women who met the recommendations. Excessive GWG introduces the mother and baby to health problems such as maternal and fetal mortality, gestational diabetes mellitus, and cesarean sections [7]. Women who gain excessive gestational weight also retain more weight postpartum thus increasing the rates of obesity after pregnancy [8, 9].

Pregnancy can be an ideal time for the adoption and maintenance of healthy lifestyle habits due to the mother’s interest in the health of her baby [7]. Although the causes of excessive GWG are multifactorial, physical activity and dietary intake are the most modifiable factors [10]. Even though previous studies have engaged pregnant women in discussions of perceived barriers to physical activity during pregnancy [11–15], to date, no such studies have been conducted among participants of the Special Supplemental Nutrition Program for Women, Infants, and Children (WIC) in Southern California. WIC women qualify for supplemental food, nutrition education, and health care referrals based on their lower incomes [16]. The geographic location of the current study is also unique because weather is a factor in the ability to exercise. Therefore, the perceptions and experiences regarding physical activity of WIC women in Southern California may be different than the perceptions and experiences of other low-income women.

Research on Latina women’s experiences in physical activity during pregnancy is limited. In a systematic review of 47 studies involving 7655 participants titled “Attitudes, barriers and enablers to physical activity in pregnant women: a systematic review,” most prior studies did not include Latina women [17]. Out of the 47 studies, we identified only 2 studies that involved focus groups with non-Black Latina women. In one study, in the 13 focus groups with 58 pregnant women, 25 (43.1%) of the participants were Latina [12]. The study was conducted in North Carolina and inquired regarding physical activity barriers, but not regarding suggested interventions [12]. A 2009 investigation included focus groups with 20 Puerto Rican and Dominican Latina women recruited from the public Obstetrics and Gynecology Clinic and Midwifery Practice of Baystate Medical Center in Massachusetts [14]. The study asked regarding barriers to physical activity but not regarding preferred interventions. Thus, prior studies using focus groups that included Latinas were published in 2009, involved a small sub-sample of Latinas, and were conducted in North Carolina and Massachusetts. Our study addresses gaps in the research by primarily focusing on Latina women in Southern California.

Focusing on Latinas’ needs is important because Latinas represent the largest racial/ethnic group in California and the country’s second-largest racial/ethnic group behind non-Latino Whites [18, 19]. In addition, Latinos are among the youngest racial/ethnic groups in the US [20]. Promoting physical activity among Latinas is especially important given that health disparities between Latinas and non-Latina Whites are persistent. For example, Latinas are more likely to develop gestational diabetes compared to African American and non-Latina White women [21]. Given the gaps in prior research, the purposes of this study are to explore the barriers that are keeping pregnant WIC women from participating in physical activity and propose interventions to increase their physical activity during pregnancy.

Given the wide reach of the WIC program, any strategies found to be effective within its population could have a tremendous impact on reducing obesity rates nationwide among this vulnerable population [16]. We explored the barriers to physical activity during pregnancy within the socioecological framework [22]. The socioecological framework explains health behaviors through several levels, including (1) intrapersonal, (2) interpersonal, (3) environmental, and (4) organizational, community, and public policy levels.

## Methods

### Design, setting of the study, characteristics of participants, and research team

Purposive sampling was used to select the study participants. A total of four in-depth focus group discussions, two in English and two in Spanish, with pregnant primarily Latina WIC women, were conducted in a private room at a WIC clinic in Southern California. FGs were held according to preferred language (English vs. Spanish-speaking) and BMI category (self-reported pre-pregnancy body weight: Normal BMI < 25 kg/m2 and high BMI ≥ 25 kg/m2). A total of four focus groups was found to be sufficient to identify a range of new issues [23]. Women were recruited if they met the study criteria of being English- or Spanish-speaking, 18 years or older, and either in the 2nd or 3rd trimester. Using a flyer created by the research team, the WIC staff from the WIC clinic in Southern California helped recruit the participants by approaching pregnant WIC participants during their WIC visit. Phone calls were made to those who expressed interest to confirm their interest and the date of the FG. Reminder calls were made 1 day before each FG. Fifty-five participants signed up to participate in the FG discussion. However, only 28 made it on the day of the FG. The reasons for no-show were not investigated. Our study complied with the Consolidated Criteria for Reporting Qualitative Research.

Four female researchers participated in this study, including a public health nutrition professor (MK), a public health professor (GDK), and two graduate students (NV and Vv). All researchers had experience in qualitative study designs, and none of the researchers had a prior relation with the FG participants.

### Focus group procedures

The FG guide was developed by the lead author (Supplementary file 1). The FG guide included questions on knowledge, attitudes, and beliefs towards exercise during pregnancy with probes to address the barriers to physical activity within the different dimensions of the socioecological framework as well as the suggested interventions to promote physical activity. The FG guide was translated into Spanish and back-translated to ensure integrity and consistency. The lead author moderated the English FGs while her graduate student took notes. The graduate student, who is bilingual and bicultural, lead the Spanish FG discussions while the lead author took notes. The number of participants in the FGs ranged from 5 to 9 for a total of 28. Before the start of the FG discussion, participants gave written informed consent and completed a questionnaire, developed by the lead author, to capture descriptive information on age, race, marital status, education, employment, number of children, number of children on WIC, number of years receiving WIC services, height, weight, pre-pregnancy weight, pregnancy due date, general health, activity level before and during pregnancy (Supplementary file 2). FGs lasted approximately 120 min. Childcare was offered at the WIC clinic, and each participant received a $25 Target gift card as a token of appreciation. Refreshments were provided for all FGs. This study was approved by the Institutional Review Board of the California State University, Fullerton.

### Analysis

SAS version 9.4 (SAS Institute, Cary, North Carolina, USA) was used to analyze the descriptive data. The methodology used to analyze the information provided by the FGs was thematic analysis. FG conversations were digitally recorded and transcribed verbatim in their respective language. Transcripts were not returned to participants for comments. Transcripts were analyzed using ATLAS. ti version 7.5. (ATLAS. ti Scientific Software Development GmbH, Berlin, Germany). Structural coding was used to classify barriers and interventions. Thematic codes for barriers and interventions were developed as each transcript was analyzed. After codes were developed, they were grouped into categories. The levels within the socioecological framework were used to represent the categories. The transcripts were independently reevaluated and compared to the existing codes and categories by the lead author and her graduate student. Disagreements in coding were discussed in research meetings until there was an inter-coder agreement.

## Results

### Characteristics of participants

Participants’ characteristics are summarized in Table 1. The mean age of FG participants was 28.9 years (SD = 6.6). Participants had been on WIC for a mean of 4.2 years (SD = 4.5). Fourteen point 3 % of the participants were self-employed or employed for wages, 50% were homemakers, and 35.6% were not working at the time of the study. Seventy-nine percent of the participants were Latina, 11% were White, and the rest self-identified as either mixed-race or other.

**Table 1:** Characteristics of focus group participants, *N* = 28

	Preferred Language		Measured pre-pregnancy BMI		Total number *n* = 28
	English *n* = 16	Spanish *n* = 12	Overweight/Obese *n* = 14	Normal Weight *n* = 14	
**Measured pre-pregnancy body mass index (BMI)**					
Normal (18.5–24.9 kg/m^2^)	9	5			14
Overweight or Obese (> 25 kg/m^2^)	7	7			14
**Gestational age (in weeks)**	25.1	30.2	28.5	26.8	28
**General health at the time of the survey**					
Excellent	5	1	3	3	6
Very good	5	4	6	3	9
Good	6	4	4	6	10
Fair	0	3	1	2	3
Poor	0	0	0	0	0
**Age**					
18–24 years	5	2	2	5	7
25–29 years	7	3	6	4	10
30–35 years	2	4	4	2	6
36+ years	1	3	1	3	4
Refused to answer	1	0	1	0	1
**Marital status**					
Married	12	5	9	8	17
Divorced	0	2	1	1	2
Separated	0	1	1	0	1
Never married	4	4	3	5	8
**Education**					
Up to 8th grade	0	2	1	1	2
9th to 11th grade	3	1	3	1	4
High school graduate or equivalent	3	5	6	2	8
Some college	7	0	1	6	7
Associate Degree	1	0	1	0	1
Bachelor’s Degree	2	0	2	0	2
No schooling Completed	0	4	0	4	4
**Preferred language**					
English			7	9	16
Spanish			7	5	12
**Activity level before pregnancy (number of times a week of 30 min or more of exercise such as walking, swimming, cycling, dancing, or gardening) (mean ± standard deviation)**	3.0 ± 2.2	3.2 ± 2.2	2.8 ± 2.1	3.4 ± 2.2	3.1 ± 2.1
**Activity level during pregnancy (number of times a week of 30 min or more of exercise such as walking, swimming, cycling, dancing, or gardening) (mean ± standard deviation)**	2.4 ± 1.9	3.0 ± 1.5	2.5 ± 1.7	2.8 ± 1.9	2.6 ± 1.8

### Barriers

#### Intrapersonal

All English-speaking overweight **(ESO)**, Spanish-speaking normal weight **(SSN)**, and Spanish-speaking overweight **(SSO)** participants reported having an intrapersonal barrier. Among the English-speaking normal weight **(ESN)** participants, 66.7% (*n* = 6) reported having an intrapersonal barrier (Table 2).

**Table 2:** Barriers to physical activity by focus group type

	Sample Size (*n* = 28)	Intrapersonal Barriers (%)	Interpersonal Barriers (%)	Environmental Barriers (%)
*Focus Group Types*				
English Normal Weight	9	66.7% (6)	77.8% (7)	11.1% (1)
English Overweight	7	100% (7)	85.7% (6)	0
Spanish Normal weight	5	100% (5)	80% (4)	0
Spanish Overweight	7	100% (7)	100% (7)	14.3% (1)

Table 3 specifies the number of participants reporting each barrier to physical activity. The most commonly cited barriers were fatigue and lack of energy Participants stated: “*I was very fatigued during my first trimester”* **(ESO)** and “*I don’t have much energy”* **(SSN)**. Other barriers included pain and swelling. For example, a participant stated: *“You could feel it after a certain amount of time that your feet are starting to swell. And then when you walk, it hurts. So, that’s the thing that’s during pregnancy that keeps you from being active”* **(ESO)**. Other commonly reported barriers were lack of child care and medical restrictions along with concerns over safety. Examples of statements included: *“Not everyone has the time and like here they provide childcare for people who have children, but if you don’t have that service then you can’t [exercise] much”****(SSO)***; *“I have to take stairs up and carrying bag after bag up the stairs … after a few trips, I noticed I was getting some pains on my side … I don’t want to hurt the pregnancy, or hurt myself, or overexert”* **(ESO)**; *“Since I’ve been pregnant, I have a fear of falling. I have this fear all the time, so I’m always careful of stepping down from where I need to step down”* **(SSO)**.

**Table 3:** Barriers according to the social ecological model

	ESN	ESO	SSN	SSO	Total
**Intrapersonal**					
*Fatigue and lack of energy*	2	8	5	5	20
*Pain and swelling*	3	8	2	2	15
*Lack of child care*	6	0	7	1	14
*Medical restrictions and safety concerns*	3	7	1	2	13
*Lack of knowledge about exercise safety*	3	3	1	2	9
*Lack of time*	0	3	0	2	5
**Interpersonal**					
*Concerns and lack of social support from partners and family members*	7	4	2	10	23
*Conflicting advice from friends and neighbors and lack of advice on safe exercise from physicians*	2	4	2	2	10
**Environmental**					
*Weather*	1	0	0	1	2

Lack of exercise knowledge was also reported as a barrier to physical activity during pregnancy. One participant said, *“For instance, a lot of pregnant people do yoga like you were saying. There’s some yoga things that you shouldn’t be doing, like when you’re lifting like your hands above your head too much,”* and another participant responded with, “*Because that means the umbilical cord are wrapped around the head”* **(ESO)**. English-speaking overweight and Spanish-speaking overweight participants cited lack of time as a barrier to physical activity*.* A participant stated, *“I was not exercising because I worked in a store, and that is a lot of walking, and then I had no time for anything. I came home until six, or at seven, I was just coming out of work... I came home to prepare all the meals for the children, clean, and get ready for another day”* **(SSO)**, and another participant added, *“The hardest part was when I could no longer do [exercise] because I had to work. It was my job or exercise; I had to work*” **(SSO)**.

#### Interpersonal

All **SSO** participants reported having an interpersonal barrier. Among the other participants, 80% (*n* = 4) of the **SSN** group, 85.7% (*n* = 6) of the **ESO** group, and 77.8% (*n* = 7) of the **ESN** group reported having an interpersonal barrier (Table 2).

Issues related to concerns and lack of social support from the participants’ romantic partners and families were the most commonly mentioned barriers to women engaging in physical activity. A participant mentioned that her husband doesn’t allow her to go out *“He doesn’t let me go out either. Almost since I became pregnant there was no way to do it. I do not vacuum, mop, I don’t even clean the bathroom. Only food, I clean my room and I lay down”* **(SSO)**. A participant said her husband prefers that she does not exert herself even by walking for the purpose of exercising, “*My husband tells me that in reality I should not walk”* **(SSN)**. Other participants mentioned their husbands did not want them to engage in physical activity due to the risk of miscarriage: *“He doesn’t want me to lift anything because he thinks I’m going to lose the baby and that scares him”* **(ESN)**. Two participants in the **SSO** group and one participant in the **ESN** group mentioned they needed to hide their exercising from their partners.

Other family members also expressed concerns and did not offer social support when it came to physical activity: *“I relate to her how she said like if you’re disabled. I can’t carry a bag of chips. I can’t carry anything at all. My mom doesn’t let me carry anything, and my boyfriend is very superstitious like when I’m eating, I have to sit up like perfectly. I can’t cross my legs. I have to sit into the car in a certain way. He has to open or close the door for me. Everything*” **(ESN)**;*“ It happens to me on my first weeks because I had a miscarriage one year before. And my mom and my sister, they don’t let me move around”* **(ESN)**. Various participants mentioned grandmothers as a barrier. One participant stated, *“She thinks that something’s going to go wrong, heart rate problems, thinks that something’s going to be wrong with the baby and that I’m doing too much”* **(ESN)**. The husband’s family was a common barrier within the **ESN** group. For example, one woman stated, *“Well, his side of the family wants me to just sit. We went to a birthday party. They’re like, “Sit, sit, sit.“ I’m like, “I’m okay. I’m okay”* **(ESN)**. Another participant mentioned her mother-in-law as a barrier: “*You shouldn’t mop. That’s not good for the baby”* **(ESO)**.

Other reported barriers included inaccurate advice from friends and neighbors and a lack of detailed information on exercise from physicians. Referring to a family friend, one participant said: “*I’ve heard a couple of times you shouldn’t be doing anything. You should be on the couch with your feet up the whole time”* (**ESO)**. One woman said she enjoyed playing soccer before getting pregnant. However, her physician told her: *“No soccer, no running, no nothing. Just walking. That’s fine. That’s safe”* (**ESN)**.

#### Environmental

Among the participants, 14.3% (*n* = 1) of the **SSO** group and 11.1% (*n* = 1) of the **ESN** group reported having an environmental barrier. The only environmental barrier that was cited was the weather. For example, the **ESN** participant cited a decline in physical activity due to hot weather. The **SSO** participant mentioned that cold weather prevented her from exercising.

#### Suggestions for physical activity interventions

We concluded our focus group sessions by asking our participants about intervention ideas pertaining to physical activity for pregnant women. Participants from all four groups mentioned wanting a walking program because they felt it is the safest form of exercise. For instance, one woman stated, *“I definitely wouldn’t want to be running, but I mean other activities, like yoga, that’s for pregnancy or other exercises that could work well. Because right now, I just feel like walking is all I do because I know that that’s safe and easy to do. But if there was somebody out there to give me more ideas, I would definitely take those ideas and try them”* **(ESO)**. Women from the **ESO** and **SSN** groups emphasized the need for more information on safe exercise. For example, one participant stated, “*I’d probably like a program that would give me more ideas that were safe for pregnancy”* **(ESO)**. Participants from all four groups expressed that a community-based program, designed specifically for pregnant women who are experiencing similar challenges to what they are experiencing, would motivate them to exercise: *“For me, as you said, to have more pregnant women with you. They can feel what you feel like. They can walk the same amount that you walk. They can eat the same kind of foods”* **(ESO)**.

Women in the **ESO** group expressed wanting health information to be delivered by doctors or older women with more experience. One woman stated, *“I think from a doctor. You feel more comfortable because you know they’re educated”* **(ESO)**. Women in the **SSN** group mentioned wanting a woman instructor. Participants in the **ESN** and **ESO** groups discussed a need for a brochure with general tips and exercise to do at home, along with tips on safe exercise and how to overcome fatigue. For example, one participant stated, “*It would be nice if they had like pamphlets or something with the certain exercises you can do at home. Little reminders like drink water, do this or do that. Just little added tips or information you can always refer back to”* **(ESO)**.

Opinions differed regarding childcare. Participants in the **ESN** and **SSN** groups mentioned needing childcare. For example, one woman stated, “*Not just for us but for our children to get a break from us too. Because believe it or not, they don’t like to be around us all the time”* **(ESN)**. All participants in the **ESO** and **SSO** groups felt strongly about including their children and families in the exercise program. They felt that including children in the exercise program would instill positive exercise behavior in them. One participant stated, “*I think family activities are better because you instill the kids to be active, so they won’t be glued to a cellphone or tablet*” **(SSO)**.

Participants in the **SSN** group mentioned the need for an exercise video featuring pregnant women. They believed the video would be good because it would motivate them. For example, one woman stated, *“For me, it would be good if they could give us a video that one can take home. There you can get motivated”* **(SSN)**. On the other hand, participants from the **SSO** group mentioned that they did not want an exercise video. They felt that an in-person class would be more motivating than a video. For example, one participant explained, “*Well with a live instructor, like now that you are talking, someone to motivate me;” and “Yes, because it’s one thing for them to tell you to do it, but when there is a person there telling you how to do it and like that, it motivates you to want to or that you won’t be alone and I don’t know if I’m doing it right or if I’m doing it wrong”* **(SSO)**.

## Discussion

This study explored the barriers to physical activity as well as interventions to promote physical activity during pregnancy among WIC participants. The World Health Organization (WHO) defines physical activity as “any bodily movement produced by skeletal muscles that require energy expenditure” [24]. Fatigue and lack of energy, pain and swelling, lack of childcare, medical restrictions and concerns over safety, lack of knowledge about exercise safety, lack of time, concerns and lack of social support from husbands and families, conflicting advice from friends and neighbors, and lack of advice from physicians were the most commonly mentioned barriers to exercising among our participants (Table 3). The most commonly proposed intervention was a community-based program where pregnant women can mingle with each other and share their challenges and concerns. Our study is unique because low-income, primarily Latina pregnant women from Southern California, identified both barriers and interventions. In the current study, fatigue, lack of energy, and lack of time were barriers to physical activity. Previous studies explained that the reasons for fatigue, lack of energy, and lack of time, in general, were due to the multiple roles that women played as caretakers of their homes, children, and other family members as well as being employed and taking a role in the workforce [12, 14, 25, 26]. It is, therefore, necessary to take into consideration the lack of time when designing physical activity interventions for pregnant women.

Our participants were fearful that exercising might hurt the baby. Such fear was likely due to the lack of knowledge about the safety of exercise [13, 27–29]. This fear was also sensed when women suggested a walking program as a physical activity intervention. They notably indicated that a walking program would be the safest. The fear of harming the baby due to false knowledge can cause women to avoid exercising during their pregnancy. Women agreed that if providers made suggestions on safe exercise, more women would feel reassured and motivated to exercise. This finding is consistent with previous research suggesting that the clinician plays an essential role in whether a pregnant woman chooses to exercise [13]. Surprisingly and unlike a previous study [13], there was no mention, during any of the focus group discussions, of the benefits of pregnancy exercise for the developing baby. This could be due to a lack of knowledge and awareness among our participants of the potential benefits of exercise for the baby. Pregnancy is an ideal time for the adoption of healthy lifestyle habits given the mother’s increased awareness about the health of her baby [7]. Therefore, interventions including specific messages that stress the benefits for the baby may encourage women to be more active during pregnancy.

Parallel to previous studies [14, 27, 30, 31], relatives and friends played a substantial role in the women’s perception of risk and engagement in physical activity. Partners and some family members were described as being overly protective, preventing women from engaging in physical activity during pregnancy. Our participants also reported receiving inaccurate advice from friends and family. In the focus groups conducted by Evenson et al. [12], the main interpersonal barrier discussed was lack of social support, especially among Latina women [12]. Therefore, interventions that engage partners might have a higher chance of success.

Our study provides directions for developing interventions to promote physical activity during pregnancy. Women in all four groups proposed having a community-based intervention where they can mingle with other pregnant women and share their challenges and concerns, which makes sense as being a part of a community has been reported to be an important factor within the Hispanic culture [32]. Other suggestions and ideas on physical activity interventions during pregnancy differed among the different groups and reflected the women’s experiences. For example, it was remarkable to see the difference when it came to childcare. All women in the overweight groups wanted to include children in exercise activities because, as they clearly stated, they wanted to instill healthy behaviors in them. Women in the normal weight group preferred to exercise without their children. Another example is the English-speaking participants wanting exercise brochures, whereas the Spanish-speaking participants preferred having a video over a brochure. These examples are good reminders that pregnant women know best what they need to help them exercise and future interventions need to be informed by these and other focus groups. Future work should also involve educating health care providers about the benefits of physical activity during pregnancy and encouraging them to go beyond telling their pregnant patients to exercise but rather reassure pregnant women and their partners by discussing safe exercises with them. A list of examples of safe and unsafe exercise along with a list of contraindications to aerobic exercise during pregnancy and warning signs to discontinue exercise during pregnancy can be found in the latest American of Obstetricians and Gynecologists publication [2].

Concerns that regular physical activity during pregnancy may cause miscarriage, poor fetal growth, musculoskeletal injury, or premature delivery are prevalent among obstetric care providers. For uncomplicated pregnancies, these concerns have not been validated [33–36]. Therefore, getting over these concerns is an essential first step. Also, future research is needed to develop the resources necessary to assist health care professionals in counseling pregnant patients regarding physical activity. For example, research on the most effective behavioral counseling and the optimal intensity and frequency of exercise during pregnancy is essential to equip health care providers with the skills and knowledge needed to support pregnant women so that they can feel more confident and at less risk when engaging in physical activity.

This study is not without limitations. The generalizability of this study may be limited, as the women were low-income WIC participants from Southern California who volunteered to participate in these focus groups. Women were eligible to participate if they were in their 2nd or 3rd trimester. Barriers to physical activity may change throughout pregnancy, so the rationale was that by including women in their 2nd and 3rd trimester, we would be able to learn about first, second, and third trimester experiences. However, there is always a possibility that participants might have forgotten about their first trimester experiences. In addition, the lack of information on the nativity of participants is a study limitation. Another limitation is that we did not use Cohen’s kappa to verify inter-rater reliability. However, team members made sure to perform reconciliation of any coding disagreements by regularly discussing these disagreements and reaching an agreement. Despite these limitations, this study adds significant information to the body of literature examining barriers to physical activity among pregnant WIC participants in Southern California, a predominately Latino population, as previous studies called for more research among minority ethnic groups [5, 11, 12, 15].

## Conclusions

This study provided insights into intrapersonal and interpersonal factors that may explain the rationales of WIC participants for making decisions related to physical activity during pregnancy. Some recommended interventions were consistent among all focus groups, and others differed by the obesity status and preferred language of women. Pregnant women know best what they need to help them exercise and future interventions need to be informed by our and other focus groups.

## Supplementary Information

**Additional file 1.** Focus Group Guide.

**Additional file 2.** Demographic Questionnaire.

## Data Availability

The datasets generated and/or analysed during the current study are not publicly available since the participants of this study did not agree for their data to be shared publicly. However, data are available from the corresponding author upon reasonable request.

## Abbreviations

FG: Focus group
ESO: English-speaking overweight
SSN: Spanish-speaking normal weight
SSO: Spanish-speaking overweight
ESN: English-speaking normal weight
