# Supplementary material for: Perceived barriers to and suggested interventions for physical activity during pregnancy among participants of the Special Supplemental Nutrition Program for Women, Infants, and Children (WIC) in Southern California
Source: BMC Pregnancy Childbirth. 2021 Jan 21;21:69. doi: 10.1186/s12884-021-03553-7 (PMC7819194; doi:10.1186/s12884-021-03553-7)
Supplement: Supplementary file 1 — Additional file 1. Focus Group Guide. [file 12884_2021_3553_MOESM1_ESM.docx]

Focus Group Guide

Fit Mommy, Healthy Baby Project

Introduction: “We would like your opinion about exercise during pregnancy. As a WIC participant, your opinion is very important to us. We want to understand how we can help mothers to be healthy during their pregnancy. Please answer the questions honestly and to the best of your ability. There are no right or wrong answers. We are only here to gather information. Our main goal is to hear about your opinions and experiences so we can develop programs to help WIC mothers be healthy during pregnancy.” Your participation in this study or anything you say during the focus group discussions will not in any way affect any WIC services or benefits you receive.

# GENERAL DISCUSSION ON HEALTH

In our session today, we will be talking about exercise and how we can be healthy during pregnancy. Let’s begin with talking about health.

1. To you, what does it mean to be healthy?
2. Generally, how healthy do you think you are? would you say you are in excellent, very good, fair or good health?
   1. Probe: why do you say you are in ________health? [ask participants to elaborate on answer]
3. During pregnancy, do you think you are as healthy as when you are not pregnant?
   1. Probe [ask participants to elaborate on answer]
4. What are you doing to be healthy, for you and your baby?
   1. Probe: do you find it hard to ___________ [repeat what participant answered in 1)? [gauge what participants say and probe from their answer]

# KNOWLEDGE, ATTITUDES, BELIEFS REGARDING EXERCISE DURING PREGNANCY

Now let’s talk about exercise during pregnancy.

1. How would you define exercise?
2. What are your opinions on exercising while pregnant?
   1. Probe for specifics such as safety.
3. In your opinion, what is the purpose of exercising during pregnancy?
   1. Probe for specifics
4. In your opinion, how much exercise is enough during pregnancy?
   1. Probe: allow participants to talk; if silent, provide examples.
5. Do you have concerns about exercising during pregnancy?
   1. Probe: if concerned, probe for specific concerns.
6. Does anyone in your family share concerns about you exercising while pregnant?
   1. Probe: allow participants to talk; if silent, offer spouse/partner/mother/mother-in-law/other. If spouse/partner/mother/mother-in-law/other has concern, probe for specific concerns raised.
7. In general, what are your family’s or significant other’s views on exercising during pregnancy?
8. Where do you receive your information on exercise during pregnancy?
9. Did your doctor, nurse, WIC staff give you advice about exercising during pregnancy?
   1. Probe: what did they say? Duration/types/safety

# BARRIERS TO EXERCISE DURING PREGNANCY

1. What are some of the reasons that are stopping you from exercising or exercising more?
   1. Probe: Time/Health/Family/ Knowledge/Motivation/ Healthcare Provider/Environment
   2. Probe for specifics
   3. Probe: how do you think you can overcome that?
   4. Probe: Did anyone tell you shouldn’t exercise or exercise less? Who?
2. Of all the things we talked about that prevent you from exercising, which one influences you the most?
   1. Probe: ask participants to elaborate on answer.

# MOTIVATORS FOR EXERCISE DURING PREGNANCY

1. What are some factors that would help you to exercise or exercise more?
   1. Probe for specifics
2. Of all the things that would help you to exercise, which ones are the most influential?
   1. Probe: ask participants to elaborate on answer.

# IDEAS FOR HELPING PREGNANT MOMS TO EXERCISE

1. Of all the things we discussed today, what do you feel is the most important?
2. In your opinion, what kind of exercise would pregnant WIC moms enjoy?
3. If you were to give advice to a friend or family member about exercise during pregnancy, what would it be?
4. If you had to choose one factor that most influences your ability to exercise, what would it be? Why?
5. In your opinion, if we are to develop a program to help pregnant moms stay active, what should it include?
   1. Probe: What specific issues do you think we need to address.
6. How else do you think WIC can encourage and help pregnant moms to stay active during pregnancy?

# CLOSING

1. Are there any areas we haven’t covered that you feel we should consider?
2. Are there any thoughts you would like to share with us before we close this session?

Thank you very much for your time and help with the interview. [*Distribute gift card to participants + enter info in gift card log*]. Your answers are very important in our efforts to promote healthy pregnancy among WIC moms. Thank you for your assistance in this effort. If you think of any other suggestions you would like to tell us, please call or email us [provide email address and phone number].

*Take notes of things said after the recorder was turned off.*
