# Supplementary material for: Perceived barriers to and suggested interventions for physical activity during pregnancy among participants of the Special Supplemental Nutrition Program for Women, Infants, and Children (WIC) in Southern California
Source: BMC Pregnancy Childbirth. 2021 Jan 21;21:69. doi: 10.1186/s12884-021-03553-7 (PMC7819194; doi:10.1186/s12884-021-03553-7)
Supplement: Supplementary file 2 — Additional file 2. Demographic Questionnaire. [file 12884_2021_3553_MOESM2_ESM.docx]

First Name ____________________________________

*DEMOGRAPHIC INFORMATION*

Please answer the following questions about your background. This information will only be used for this project and will not be released to anyone without your written consent. Thank you for your willingness to participate in this important project to help WIC mothers and babies.

1. What is your date of birth (month/year)? _____________________________
2. What is your racial/ethnic background? (circle/check all that apply)
   1. Latina
   2. African-American
   3. Asian
   4. White
   5. American Indian
   6. Pacific Islander
   7. Other
3. What is your marital status?
   1. Married
   2. Widowed
   3. Divorced
   4. Separated
   5. Never married
4. Which of the following people live in your household? (circle/select all that apply)
   1. Baby’s dad
   2. Your mother
   3. Your mother-in-law
   4. None of the above
5. What is the highest degree or level of school you have completed? If currently enrolled, mark the previous grade or highest degree received.
   1. Up to 8th grade
   2. 9^th^ to 11^th^ grade
   3. High school graduate or equivalent (GED)
   4. Some college
   5. Associate degree (for example: AA, AS)
   6. Bachelor's degree (for example: BA, AB, BS)
   7. Master's degree
   8. Professional or doctorate degree
   9. No schooling completed
6. Are you currently_____________________________?
   1. Employed for wages
   2. Self-employed
   3. Out of work but looking for work
   4. Out of work but not currently looking for work
   5. A homemaker
   6. A student
   7. Unable to work
7. How many children do you have? (Please **DO NOT** include your current pregnancy)
   1. ____________________________
8. For how many years have you or your children received services and food from the WIC program? ________________ years.
9. How many of your children are participating in WIC currently? ____________________.
10. What is your height? __________ ft _________in
11. What is your current weight? __________________________ pounds
12. What was your weight just before this pregnancy? ____________________ pounds
13. What is your **due date** for this pregnancy? __________________________
14. Would you say that in general, your health is:
    1. Excellent
    2. Very good
    3. Good
    4. Fair
    5. Poor
15. Think about how active you were **BEFORE** you became pregnant with your baby. At that time, how many times a week did you exercise for 30 minutes or more, such as walking, swimming, cycling, dancing, or gardening;
    1. ______________ times a week
    2. Less than once a week
    3. Never
16. Think about how active you are **RIGHT NOW**. How many times a week do you exercise for 30 minutes or more, such as walking, swimming, cycling, dancing, or gardening;
    1. ________________ times a week
    2. Less than once a week
    3. Never
